# Multiscale neural signatures of long-lag repetition learning: a simultaneous fNIRS–EEG study

**DOI:** 10.1117/1.NPh.13.4.045001

**Published:** 2026-10-16

**Authors:** Jinglei Ren, Adam Noah, Xian Zhang, Vincent Gracco, Marc Joanisse, Aahana Bajracharya, Jay G. Rueckl, Augusto Buchweitz, Nabin Koirala, Joy Hirsch, Ken Pugh

**Affiliations:** a Yale School of Medicine, Yale Child Study Center, New Haven, Connecticut, United States; b Yale School of Medicine, Brain Function Laboratory, New Haven, Connecticut, United States; c The University of Western Ontario, Department of Psychology, London, Ontario, Canada; d University of Connecticut, Department of Psychological Sciences, Storrs, Connecticut, United States; e University of Connecticut, Brain Imaging Research Core (BIRC), Storrs, Connecticut, United States; f Nathan Kline Institute for Psychiatric Research (NKI), Center for Biomedical Imaging & Neuromodulation, Computational Neuroimaging Laboratories, New York, New York, United States

**Keywords:** long-lag repetition learning, multimodal neuroimaging, multiscale neural adaptation, functional near-infrared spectroscopy, electroencephalography

## Abstract

**Significance:**

Repetition-based learning supports word recognition and reading fluency, yet its neural mechanisms remain poorly understood, particularly where deficits in consolidating repeated linguistic input may underlie reading and language difficulties. Establishing reliable neural markers of repetition-based learning is a critical first step toward characterizing the phenotypes of these difficulties in children.

**Aim:**

We developed a whole-brain simultaneous functional near-infrared spectroscopy–electroencephalography (fNIRS–EEG) approach to capture fast temporal electrophysiological and spatially accurate hemodynamic signals during long-lag repetition learning.

**Approach:**

Thirty participants performed a semantic judgment task with words repeated across four runs with intervening items, modeling repetition demands central to reading instruction. A multimodal decoding framework integrating electrophysiological, hemodynamic, and cross-modal fNIRS–EEG correlation features was used to classify repetition-based learning.

**Results:**

Repeated words showed response time reductions. EEG captured a frontal N400-range repetition-related negativity and attenuation of alpha–beta suppression, reflecting changes in recognition-related and post-recognition processing. fNIRS revealed left inferior frontal and superior temporal efficiency increases alongside right supramarginal activation, suggesting integrative processing. Multimodal classifiers outperformed unimodal classifiers, indicating that each modality captured complementary aspects of learning.

**Conclusions:**

Combining fNIRS and EEG with multimodal machine learning provides a richer characterization of repetition-based learning than either modality alone, establishing a framework for phenotyping language learning capacity and identifying neural markers of reading difficulties in pediatric populations.

## 1 Introduction

Efficient word recognition and reading fluency depend critically on the brain’s capacity to learn from repeated encounters with linguistic stimuli. This capacity, repetition-based language learning, is central to vocabulary acquisition, reading development, and language instruction,^1^^,^^2^ yet its neural mechanisms remain poorly understood, particularly in the context of disorders where deficits in consolidating repeated linguistic input may underlie persistent reading and language difficulties.^3^ Understanding how the brain builds durable representations through repetition is therefore not only a fundamental question in cognitive neuroscience but also a clinically meaningful one. Identifying reliable neural markers of repetition-based learning in adults is a critical first step toward developing tools for deeper phenotyping of reading and language difficulties in children.

Dominant theoretical accounts, including representational sharpening,^4^ neural efficiency,^5^ and predictive coding,^6^^,^^7^ converge on the principle that repetition reduces the neural resources required to process a familiar item. However, this convergence belies a persistent tension in the empirical record. Alongside well-documented repetition suppression, a reduction in neural response magnitude upon stimulus re-encounter, a substantial body of evidence reports repetition enhancement in specific brain regions and task contexts,^8^^–^^11^ suggesting that repetition learning may produce dissociable neural signatures across different levels of measurement rather than a uniform pattern of neural attenuation.

This coexistence of suppression and enhancement has received competing interpretations. Suppression has been linked to reduced prediction error and increased processing efficiency,^6^^,^^7^ whereas enhancement has been associated with representational updating, consolidation, and integrative processing.^10^^,^^12^ Resolving this tension has direct relevance for understanding language learning, as the neural systems implicated in repetition suppression and enhancement, including left inferior frontal and superior temporal cortices, are canonically associated with phonological processing, lexical access, and reading^13^^,^^14^ and are known to be atypically organized in individuals with developmental dyslexia and language learning disorders.^15^^,^^16^ However, because most prior studies have relied on a single neuroimaging modality, it remains difficult to characterize how these opposing effects relate to one another across different levels of neural organization or to identify which neural signatures most sensitively capture individual differences in language learning capacity. Addressing this limitation requires two methodological advances that together form the foundation of the present work. The first is an experimental paradigm that isolates durable learning from transient facilitation. The second is a multimodal recording approach that can simultaneously index neural activity at complementary timescales.

The first requirement, isolating durable learning, points to the importance of temporal spacing between repetitions. Short-lag designs, in which repeated items occur within seconds or a few intervening trials, are strongly influenced by perceptual habituation, residual working memory activation, and short-lived predictions.^5^^,^^17^ Under such conditions, observed repetition effects may reflect transient sensory adaptation rather than enduring representational change. By contrast, long-lag repetition paradigms, with many unrelated intervening items, allow these transient influences to dissipate.^18^^–^^21^ Any repetition effect that survives such extended intervals must arise from stored representations that have been durably modified by prior experience. Behaviorally, long-lag repetition produces facilitation in response times and accuracy,^22^^,^^23^ confirming that enduring learning has occurred.

The second requirement, multimodal recording, is motivated by the complementary nature of electrophysiological and hemodynamic signals. At the electrophysiological level, long-lag repetition modulates a cascade of event-related potential (ERP) components with distinct functional associations. Repetition-related reductions have been observed in the FN400 (300 to 500 ms), a frontal negativity linked to familiarity-based recognition,^12^^,^^24^ and in the N400 (300 to 500 ms), associated with lexico-semantic retrieval demands.^25^ Their dissociable scalp distributions suggest separable neural generators. Concurrently, repeated items often elicit enhanced late positive activity (the LPC, >500  ms), linked to recollective processing.^12^^,^^26^ Time–frequency analyses further reveal repetition-related oscillatory modulations, including increased alpha power and theta synchronization, associated with representational stabilization and predictive updating.^27^^,^^28^ Together, these findings indicate that repetition engages multiple fast computations operating at distinct temporal scales. Hemodynamic signals, by contrast, index neural activity at a fundamentally different timescale: they reflect the cumulative metabolic demands of processing over seconds, integrating across fast electrophysiological events. Functional MRI studies have frequently reported repetition-related decreases in inferior frontal, superior temporal, and occipitotemporal cortices,^11^^,^^29^^–^^31^ typically interpreted as increased metabolic efficiency.

Prior work has begun to integrate across modalities. Simultaneous EEG-fMRI studies have linked electrophysiological modulations to BOLD adaptation,^32^^–^^35^ yet the constrained scanning environment limits ecological validity and introduces EEG artifacts that complicate signal interpretation. These limitations are particularly problematic for learning paradigms, which require extended task durations and naturalistic conditions to elicit durable representational change. Functional near-infrared spectroscopy (fNIRS), which measures changes in oxygenated (HbO) and deoxygenated (HbR) hemoglobin concentration in cortical tissue, offers a promising alternative: fNIRS retains sensitivity to regional hemodynamic dynamics while permitting concurrent EEG acquisition with minimal electromagnetic interference and greater participant comfort. However, existing fNIRS-EEG studies in language processing have primarily focused on short-timescale processes such as speech perception and semantic judgment,^36^^,^^37^ and very few studies, if any, have developed a whole-cortex simultaneous fNIRS–EEG framework to characterize how repetition learning unfolds over extended repetition intervals.

Machine-learning-based multimodal decoding provides a principled approach to evaluating this complementarity. Decoding models quantify the discriminative information carried by each modality and directly test whether their integration improves predictive accuracy, thereby assessing whether electrophysiological and hemodynamic signals provide complementary information about learning-related representational change.^38^^,^^39^ Because fNIRS and EEG index partially independent physiological processes, namely, rapid neural signaling and slower metabolic adaptation, multimodal decoding enables characterization of learning across multiple timescales that cannot be captured by either modality alone. In addition to modality-specific features, such models can incorporate cross-modal features indexing temporally aligned fNIRS-EEG relationships, allowing sensitivity to aspects of cross-modal fNIRS–EEG correlation features without assuming a one-to-one mapping between electrical and hemodynamic signals.

In the present study, we address this gap by developing a simultaneous fNIRS–EEG multimodal neuroimaging approach and applying it to a long-lag repetition paradigm, enabling the concurrent measurement of fast electrophysiological dynamics and slower hemodynamic responses during learning. Our design yields three levels of predictions. First, if repetition-related changes are sufficiently captured at a single physiological level, either electrophysiological or hemodynamic measures alone may be sufficient to discriminate repetition status, and combining modalities may provide little additional discriminative information. Second, if the modalities capture complementary aspects of repetition-related adaptation, they may show partially dissociable patterns: electrophysiological signals may be sensitive to rapid predictive and representational processes, including FN400/N400 and oscillatory modulations, whereas hemodynamic signals may capture slower, regionally distributed changes in cortical activity. Under this working multiscale adaptation framework, multimodal classifiers integrating electrophysiological, hemodynamic, and cross-modal fNIRS–EEG correlation features are expected to outperform unimodal models if the two modalities contain complementary discriminative information. Importantly, this framework does not assume that repetition suppression or enhancement is specific to either modality. Third, if the multimodal framework captures behaviorally meaningful learning signals rather than incidental physiological variation, individual differences in multimodal classification accuracy should correlate with the magnitude of behavioral repetition learning, such as response-time facilitation across repetitions.

## 2 Methods

### 2.1 Participants

Thirty adults participated in this study. Participants received $40 (US dollars) compensation for their time. Our sample size of 30 participants is consistent with prior simultaneous fNIRS-EEG studies from our group,^40^^–^^42^ which used comparable sample sizes (N=21 to 28) for within-subject multimodal paradigms. A post hoc sensitivity analysis (G*Power 3.1) indicated that with N=30, α=0.05, and power=0.80, the minimum detectable effect size for a paired t-test is d=0.53. The study was not pre-registered.

### 2.2 Experimental Design

Participants performed a semantic judgment task in which they indicated whether each visually presented English word referred to a living or nonliving entity. Each trial began with a fixation cross presented for 500 ms, followed by the word displayed for 2 s. The inter-stimulus interval was jittered between 0.5 and 1 s to reduce temporal predictability. Participants responded “living” or “nonliving” using the red and green buttons on an Xbox controller. Trials were organized into six-item blocks, with each block containing six fixed lexical items (e.g., ARTIST, CAT, APPLE). The order of words within each block was randomized on each presentation. Each run consisted of five blocks: four repeated blocks (R1 to R4) that reappeared across all four runs, and one novel block (N1 to N4) unique to that run that never recurred (Fig. 1). The four repeated blocks were presented once per run, resulting in three additional exposures (runs 2, 3, and 4) following their initial presentation in run 1. Four repeated blocks were included to provide multiple observations at each presentation for estimating repetition-related changes and conducting block-level classification, whereas the novel block in each run was included to characterize nonspecific changes across runs, such as practice, fatigue, and time-on-task effects. A 10 to 15 s inter-block interval was included to allow the hemodynamic response to return to baseline. Block order within each run was pseudorandomized to prevent participants from anticipating whether a given block contained novel or repeated items. Trial order was pseudorandomized within each block. Participants completed four runs in total. The organization of runs and the long-lag repetition structure are shown schematically in Fig. 1.

**Fig. 1:**
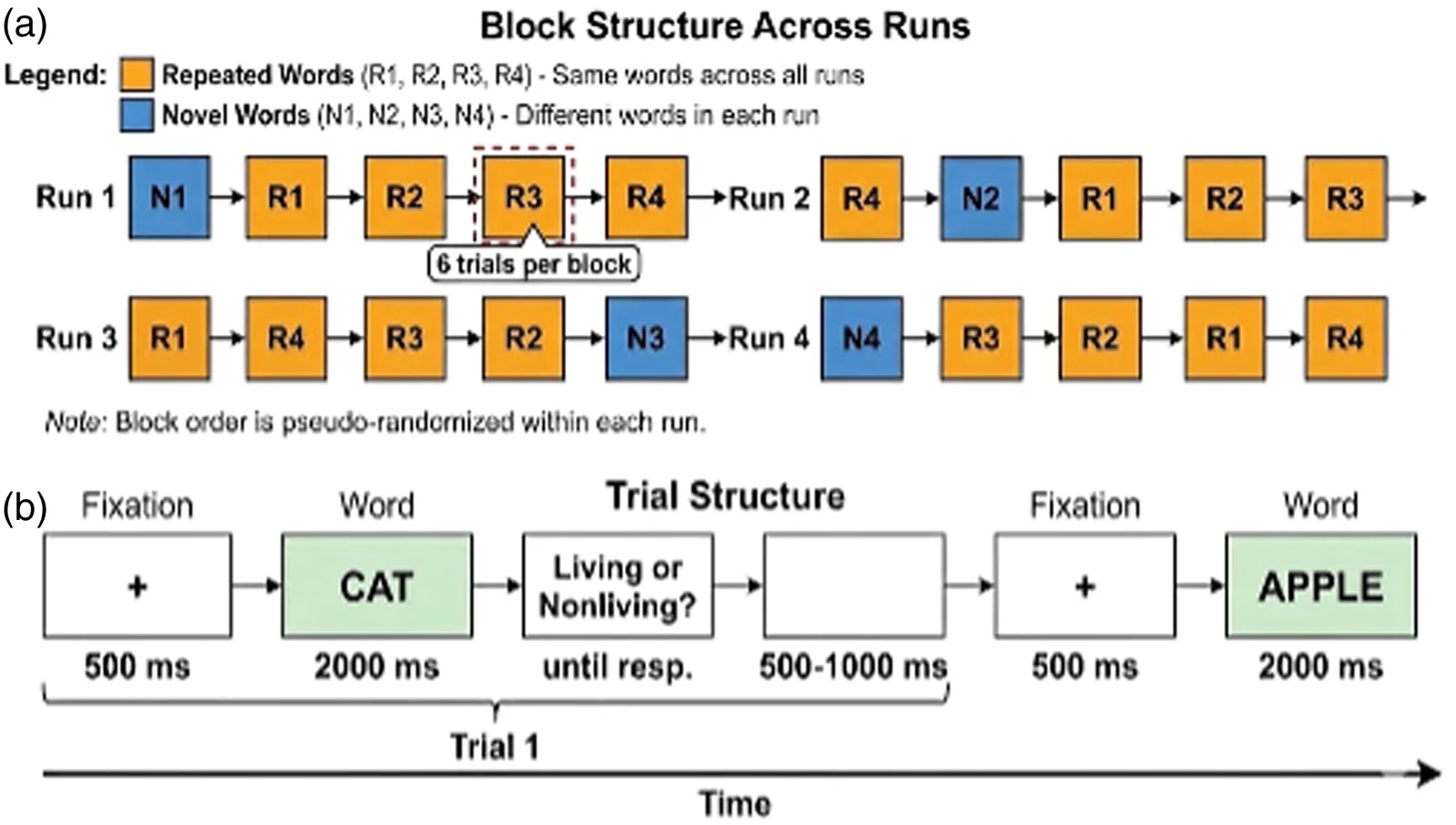
Experimental design and repetition structure of the long-lag repetition task. (a) Participants completed four runs of a semantic decision task (living/nonliving judgment). Each run consisted of five blocks, with each block containing six words. Four repeated blocks (R1 to R4, orange) appeared in all four runs, whereas each run also contained one unique novel block (N1 to N4, blue) that never recurred. Block order was pseudorandomized within each run. (b) Trial structure. Each trial began with a fixation cross (500 ms), followed by word presentation (2000 ms), response prompt, and a jittered inter-stimulus interval (500 to 1000 ms). Trial order was also pseudorandomized within each block.

### 2.3 fNIRS Acquisition

We recorded fNIRS signals using a continuous-wave LABNIRS system (Shimadzu Corporation, Kyoto, Japan) consisting of 40 emitter–detector pairs arranged in a matrix configuration yielding 134 measurement channels (Fig. 2). The fNIRS system employed three wavelengths (780, 805, and 830 nm) to measure changes in oxygenated and deoxygenated hemoglobin concentrations, with a sampling rate of 8.13 Hz (123 ms per sample). The anterior midline optode was positioned 1 cm above the nasion, the posterior midline optode aligned with the inion, and the Cz optode positioned at the anatomical Cz to ensure consistent cortical coverage across participants. Hair beneath each optode was cleared using a lighted fiber-optic probe (Daiso, Hiroshima, Japan) to optimize contact. The signal-to-noise ratio was assessed before data collection by monitoring light attenuation in each channel, and optode placement was adjusted as needed.^43^^,^^44^ Data acquisition and cap placement followed established protocols.^45^^–^^48^

**Fig. 2:**
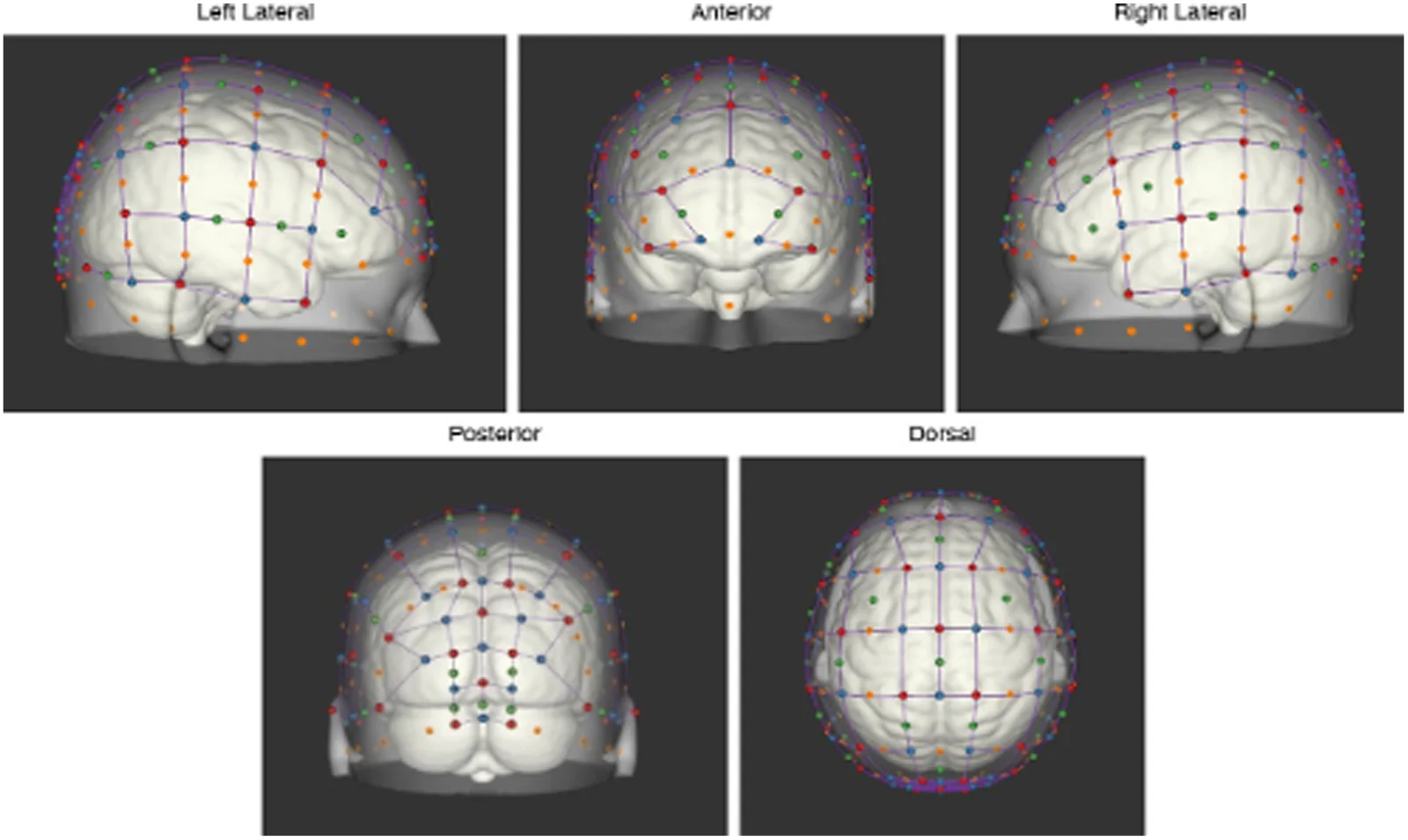
Spatial configuration of simultaneous fNIRS-EEG recording. Red spheres indicate fNIRS sources and blue spheres indicate fNIRS detectors (40 emitter–detector pairs yielding 134 measurement channels; LABNIRS, Shimadzu Corporation); green spheres indicate EEG electrode positions (32 channels arranged according to the international 10 to 10 system; g.USBamp, G.tec Medical Engineering). The fNIRS optode array was positioned with the anterior midline optode 1 cm above the nasion, the posterior midline optode aligned with the inion, and the Cz optode at anatomical Cz to ensure consistent cortical coverage across participants. EEG electrode placement was adapted to accommodate the fNIRS optodes.

### 2.4 EEG Acquisition

We recorded EEG data simultaneously with fNIRS using a 32-electrode, 256 Hz system (g.USBamp, G.tec Medical Engineering, Austria). Electrodes were arranged according to the international 10 to 10 system, adapted to accommodate the fNIRS optodes (Fig. 2). Cz served as the online reference and was excluded from subsequent analyses, yielding 31 scalp channels. The recording montage did not include mastoid electrodes due to spatial constraints imposed by the simultaneous fNIRS optode placement. After cap placement, saline-based conductive gel was applied to each electrode to ensure scalp contact. Signal quality was visually inspected using an oscilloscope to verify stable waveforms and absence of excessive noise or saturation. Electrode impedances were confirmed via the amplifier interface and maintained below 10 kΩ throughout the recording.

### 2.5 Optode and Electrode Localization

After completion of the task, we recorded three-dimensional coordinates of all optodes and electrodes using a Structure Sensor 3D scanner (Structure.io, Boulder, Colorado, United States) to create a participant-specific scalp model.^49^^,^^50^ Standard cranial landmarks (nasion, inion, Cz, T3, T4) were manually identified, and electrode positions were defined as the midpoints between adjacent optodes. Coordinates were then normalized to Montreal Neurological Institute (MNI) space^51^ using NIRS-SPM.^52^ Estimated spatial accuracy of this probabilistic registration approach is ∼10 to 15 mm.^49^

### 2.6 fNIRS Preprocessing and Statistical Analysis

Preprocessing was conducted in NIRS Brain AnalyzIR Toolbox^53^ and MATLAB R2024a. Raw optical density signals were converted to relative oxyhemoglobin (OxyHb) and deoxyhemoglobin (deOxyHb) concentration changes using the modified Beer–Lambert law.^54^^,^^55^ Baseline drift was removed using wavelet detrending,^56^ and global physiological noise was attenuated using a PCA-based spatial filter.^57^^,^^58^ PCA-based filtering is not a substitute for short-separation regression. During data acquisition, channel quality was assessed by monitoring detected-light attenuation and detector saturation. Hair was displaced, and optode positions were adjusted as needed until stable signal intensity was obtained. Quality control was therefore performed primarily during acquisition rather than by applying a fixed numerical post hoc channel-rejection threshold. Following wavelet detrending, PCA-based spatial filtering was applied separately to the multichannel HbO and HbR signals following the procedure described by Zhang et al. (2016, 2017).^57^^,^^58^ The data were decomposed using singular value decomposition, and the spatial loading pattern of each component was smoothed using the broad Gaussian kernel specified in the original method. The spatially broad components were used to reconstruct an estimate of the global signal, which was then subtracted from the original data. Thus, no fixed number of principal components was removed. We also clarified that, although this procedure can attenuate spatially widespread physiological fluctuations, it cannot fully distinguish cortical activity from superficial scalp blood flow or spatially heterogeneous systemic effects because short-separation channels and concurrent systemic physiological measurements were not available.

A general linear model (GLM^59^) was implemented to estimate hemodynamic responses for each of the four presentations of repeated words (runs 1 to 4) and novel words (runs 1 to 4). Eight boxcar regressors modeling repeated-word and novel-word presentations in runs 1 to 4 were constructed based on event timings and convolved with the canonical hemodynamic response function from the NIRS Brain AnalyzIR toolbox. The GLM was fitted per participant per channel, yielding run-specific beta estimates (β_Run1, β_Run2, β_Run3, and β_Run4) for each condition at each of the 134 measurement channels. Analyses were performed on an HbDiff signal, computed as the sum of the absolute concentration changes in OxyHb and deOxyHb, which provides a more robust index of cortical activation and reduces susceptibility to false positives in fNIRS measurements.^60^ However, HbDiff does not preserve the direction or canonical relationship of HbO and HbR and therefore cannot identify their separate contributions.

For the primary analysis, we computed first-versus-last-run contrasts for repeated words. Specifically, paired t-tests compared β_Run1 versus β_Run4 at each channel across participants (random effects, df=N−1), separately for the run 1 > run 4 and run 4 > run 1 directions. To characterize the time course of repetition effects, we also examined pairwise contrasts across successive runs (run 1 versus run 2, run 2 versus run 3, run 3 versus run 4) for both repeated and novel conditions. Novel words served as a control condition to confirm that observed effects were repetition-specific rather than attributable to general practice or fatigue. All channel-wise tests were corrected for multiple comparisons using false-discovery rate (FDR) correction with q<.05 across all 134 channels.^61^

Statistical inference was conducted at the channel level; no spatial normalization of functional data was performed. MNI coordinates were used solely for anatomical labeling and visualization. After data collection, optode positions were digitized with a Structure Sensor 3D scanner, registered to MNI space via NIRS-SPM^52^ using the Colin27 template and standard cranial landmarks, and each channel was assigned an anatomical label based on the cortical projection of the midpoint between its source and detector. The estimated spatial accuracy of this probabilistic registration is ∼10 to 15 mm.^49^

### 2.7 EEG Preprocessing and Statistical Analysis

EEG preprocessing was performed using EEGLAB 2021^62^ and MNE-Python 1.11.^63^ Continuous data were band-pass filtered (1 to 100 Hz). Bad channels were identified via automated criteria (median absolute deviation >3 relative to the channel median, and/or mean inter-channel correlation <0.3) and interpolated using spherical splines. Data were re-referenced to the common average. Independent component analysis (ICA; extended infomax, 20 components) was applied, and components with <5% probability of neural origin as classified by ICLabel,^64^ or components representing eye or muscle artifacts, were excluded. Only correctly responded trials were included in the following whole-scalp and time-frequency analyses.

For whole-scalp analysis, cleaned continuous data were band-pass filtered (0.1 to 30 Hz), epoched from −200 to 800 ms relative to word onset, and baseline-corrected using the −200 to 0 ms pre-stimulus interval. Epochs exceeding ±80  μV on any channel were rejected (M=2.8%, range: 0% to 14.2%). The primary ERP contrast compared the first (run 1) versus fourth (run 4) presentation of repeated words. Spatio-temporal cluster-based permutation tests^65^ were conducted across all channels and all post-stimulus time points (0 to 800 ms), with 10,000 permutations and a cluster-forming threshold of p<.05 (two-tailed). This whole-scalp, data-driven approach eliminates the need for *a priori* ROI or time-window specification and directly addresses multiple comparison concerns. As secondary analyses, we tested the linear trend across all four runs using orthogonal contrast weights (−3,−1,+1,+3) and conducted pairwise comparisons between adjacent runs (run 1 versus 2, run 2 versus 3, run 3 versus 4). The same run 1 versus run 4 contrast was also applied to novel words to confirm that any observed effects were repetition-specific. Effect sizes are reported as Cohen’s d for all comparisons.

To complement the time-locked ERP analysis, oscillatory dynamics were examined using time-frequency decomposition. Spectral power was computed at the single-trial level using Morlet wavelet convolution (2 to 30 Hz, 1 Hz steps, adaptive cycles=frequency/2), yielding a time-frequency representation for each trial, electrode, and participant. Event-related spectral perturbation (ERSP) was quantified in decibels ($10\times \log_{10}[\text{power}/\text{baseline}]$) relative to the pre-stimulus interval (−200 to 0 ms). ERSP values were averaged across all electrodes and across trials within each run for each participant. Statistical comparison between the first and fourth presentations of repeated words was conducted using a cluster-based permutation test (10,000 permutations, cluster-forming threshold p<.05, two-tailed) applied to the full frequency × time matrix, controlling for multiple comparisons across all time-frequency points simultaneously.

### 2.8 Machine Learning Analysis

#### 2.8.1 ROI selection

To enable multimodal integration, a common set of anatomical regions was defined for both fNIRS and EEG feature extraction. Three regions of interest were selected *a priori* based on their established roles in the visual word processing network and their consistent modulation by repetition in prior fMRI studies:^11^^,^^29^^–^^31^ left inferior frontal gyrus (IFG), left superior temporal gyrus (STG), and left ventral occipitotemporal cortex (vOT). These ROIs were defined independently of the present unimodal results. For fNIRS, channels were assigned to ROIs based on probabilistic cortical projection of optode positions into MNI space. Channel-level features were averaged within each ROI. For EEG, because scalp electrodes have limited spatial precision, coarse electrode clusters were selected to approximate the same cortical territories: left frontal (F7, F3, FC5) as a proxy for IFG, left temporal (T7, CP5) as a proxy for STG, and left ventral occipitotemporal (P7, PO3, O1) as a proxy for vOT. Electrode-to-ROI assignments were based on minimum Euclidean distance between standard 10 to 10 coordinates and MNI centroids of target regions.^66^^,^^67^

#### 2.8.2 Feature extraction and aggregation

To determine whether multimodal brain features could distinguish repeated words in first versus last presentation, both fNIRS and EEG data were converted into quantitative feature matrices suitable for machine learning classification. fNIRS features were averaged within each of three ROIs (left IFG, left STG, left vOT), yielding six fNIRS features per block (2 features × 3 ROIs). EEG features were summarized over coarse scalp regions approximating the same language-related territories: left frontal (F7, F3, FC5; proxy for IFG), left temporal (T7, CP5; proxy for STG and posterior temporal cortex), and left ventral occipitotemporal (P7, PO3, O1; proxy for ventral occipitotemporal cortex associated with visual word processing). These EEG ROIs were defined *a priori* based on cortical regions implicated in word repetition learning, independent of both the whole-scalp ERP analysis and the unimodal fNIRS results. Electrode clusters were selected based on minimum Euclidean distance between standard 10 to 10 electrode coordinates and MNI centroids of target cortical regions.^66^^,^^67^

Mean amplitudes were extracted from canonical ERP windows corresponding to the early (150 to 300 ms), middle (300 to 500 ms), and late (500 to 800 ms) components, yielding nine ERP features per block (3 components × 3 regions). Spectral power in theta (4 to 7 Hz), alpha (8 to 13 Hz), and beta (13 to 25 Hz) bands yielded nine additional spectral features per block (3 bands × 3 regions). To assess cross-modal correspondence, fNIRS and EEG features were correlated within each region, generating 36 coupling features across the three regions. Principal component analysis (PCA) was applied exclusively to the coupling feature set to reduce dimensionality. For each participant and block, trial-level fNIRS and EEG features were matched by stimulus trial within each ROI, and Pearson correlations were calculated across the six trials. The two fNIRS features, mean and standard deviation of HbDiff, were each correlated with three ERP features and three spectral-power features, yielding 12 correlations per ROI and 36 correlations across the three ROIs. These values were assigned exclusively to the corresponding participant-by-block classification sample and were not computed across blocks or participants. To control for nonspecific time-on-task effects, fNIRS and EEG features were residualized for run-related drift within each LOSO fold. At the beginning of each fold, one participant and all corresponding observations were held out. Run-related drift coefficients were estimated using only the novel-word blocks from the training participants, PCA was fitted only to the training-set coupling features, and z-scoring parameters were calculated only from the training data. The fitted residualization model, PCA loadings, and scaling parameters were then applied unchanged to the held-out participant.

#### 2.8.3 Classification procedure

We used support vector machines (SVMs^68^) to classify repeated words at first (run 1) versus fourth presentation (run 4). A linear SVM with C=1 was used for all classifiers. This value was fixed *a priori*, and no data-driven hyperparameter optimization was performed using either the full dataset or the held-out participant. The same regularization parameter was used for the fNIRS-only, EEG-only, and multimodal models. The linear kernel was chosen to constrain model complexity and permit inspection of model coefficients. Each participant contributed four blocks from run 1 and four blocks from run 4, yielding eight observations per participant and 240 total across 30 participants. Classification was performed exclusively on repeated words. All models were trained and tested using leave-one-subject-out (LOSO) cross-validation. The SVM was trained only after all fold-specific transformations had been completed. For the feature count-matched analyses, features were ranked within each training fold according to the absolute standardized mean difference (|Cohen’s d|) between repeated and novel blocks. The selected feature set was then applied unchanged to the held-out participant. Thus, the held-out participant did not contribute to the estimation of residualization coefficients, PCA loadings, scaling parameters, feature rankings, selected feature sets, or model parameters. Three classifiers were evaluated: (1) an fNIRS-only model (six features), (2) an EEG-only model (18 features), and (3) a multimodal model combining unimodal features with PCA-reduced cross-modal fNIRS–EEG correlation features. For the multimodal linear SVM classifier, we summarized relative model-weight patterns using the absolute magnitudes of the learned coefficients. Because correlated predictors may share or redistribute coefficient magnitude, these values should not be interpreted as measures of the unique physiological or predictive contribution of individual features. Accordingly, the precise ranking of individual features is considered exploratory. The EEG–HbDiff correlations reflect associations between electrophysiological activity and the composite hemodynamic response, rather than HbO- or HbR-specific neurovascular coupling. Statistical significance was assessed using 1000-iteration permutation testing. Feature-importance values from the multimodal linear SVM classifier were computed as the absolute magnitude of the model’s learned feature weights. See Fig. 3 for machine learning classification pipeline for fNIRS-EEG multimodal analysis.

**Fig. 3:**
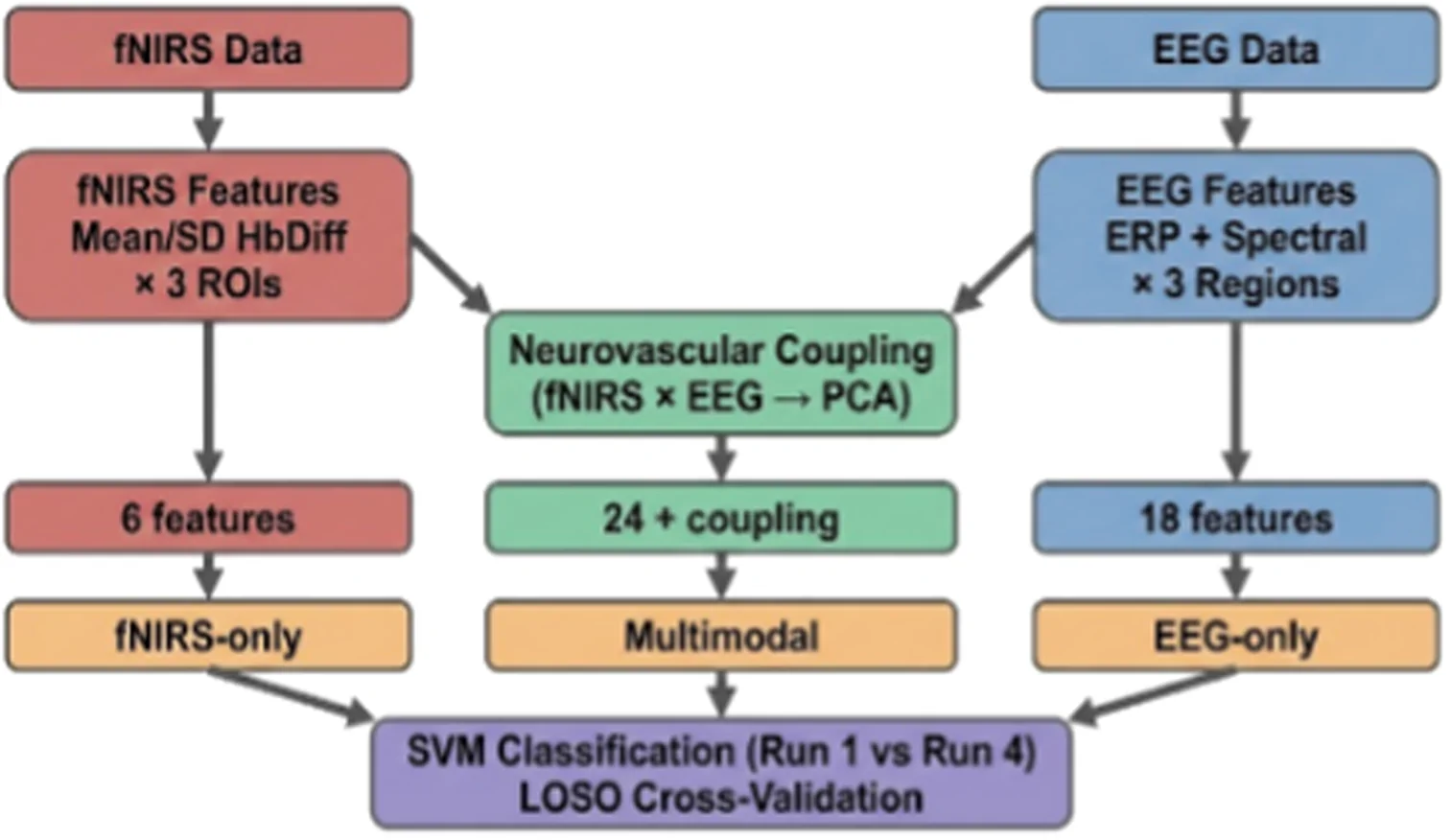
Machine learning classification pipeline for fNIRS-EEG multimodal analysis. Unimodal features: 6 fNIRS (HbDiff mean/SD × 3 ROIs) and 18 EEG (ERP + spectral × 3 regions). Cross-modal correlation features derived from fNIRS-EEG correlations were reduced via PCA. SVM classifiers with LOSO cross-validation distinguished run 1 versus run 4 of repeated words.

## 3 Results

### 3.1 Behavioral Results

Accuracy was near ceiling across all conditions and runs (repeated: 98.0%; novel: 95.2%), and was not further analyzed. Reaction time (RT) data were analyzed for 26 participants (four participants were excluded due to missing triggers: subjects 4, 8, 19, and 27). To test whether RT changes were repetition-specific, we examined the condition × run interaction. The RT reduction from run 1 to run 4 was significantly larger for repeated words (−105  ms) than for novel words (−40  ms), confirming a repetition-specific facilitation effect (t=−2.49, p=.021, d=0.53). A repeated-measures ANOVA with run (1 to 4) as the within-subject factor revealed a significant main effect of repetition for repeated words, F(3,78)=2.894, p=.039, indicating that RTs differed significantly across the four runs. As shown in Fig. 4(a), RTs were longest in run 1 (M=0.894  s, SD=0.144) and progressively decreased across run 2 (M=0.828  s, SD=0.146) and run 3 (M=0.806  s, SD=0.170), reaching the fastest responses in run 4 (M=0.776  s, SD=0.138). Pairwise comparisons demonstrated robust repetition-related speeding. Relative to the first exposure, responses were significantly faster in run 2, t=5.022, p<.001, d=0.985, run 3, t=4.326, p<.001, d=0.848, and run 4, t=7.289, p<.001, d=1.429. Direct comparisons between intermediate runs also showed significant improvements (run 2 versus run 4: t=3.575, p=.001, d=0.701; run 3 versus run 4: t=2.468, p=.021, d=0.484), confirming progressive RT facilitation with repeated exposure. For novel words, no significant main effect of run was observed (F<1, p=.55). Participants responded faster in run 2 compared with run 1 (t=2.180, p=.039, d=0.430), but other direct comparisons were not significant.

**Fig. 4:**
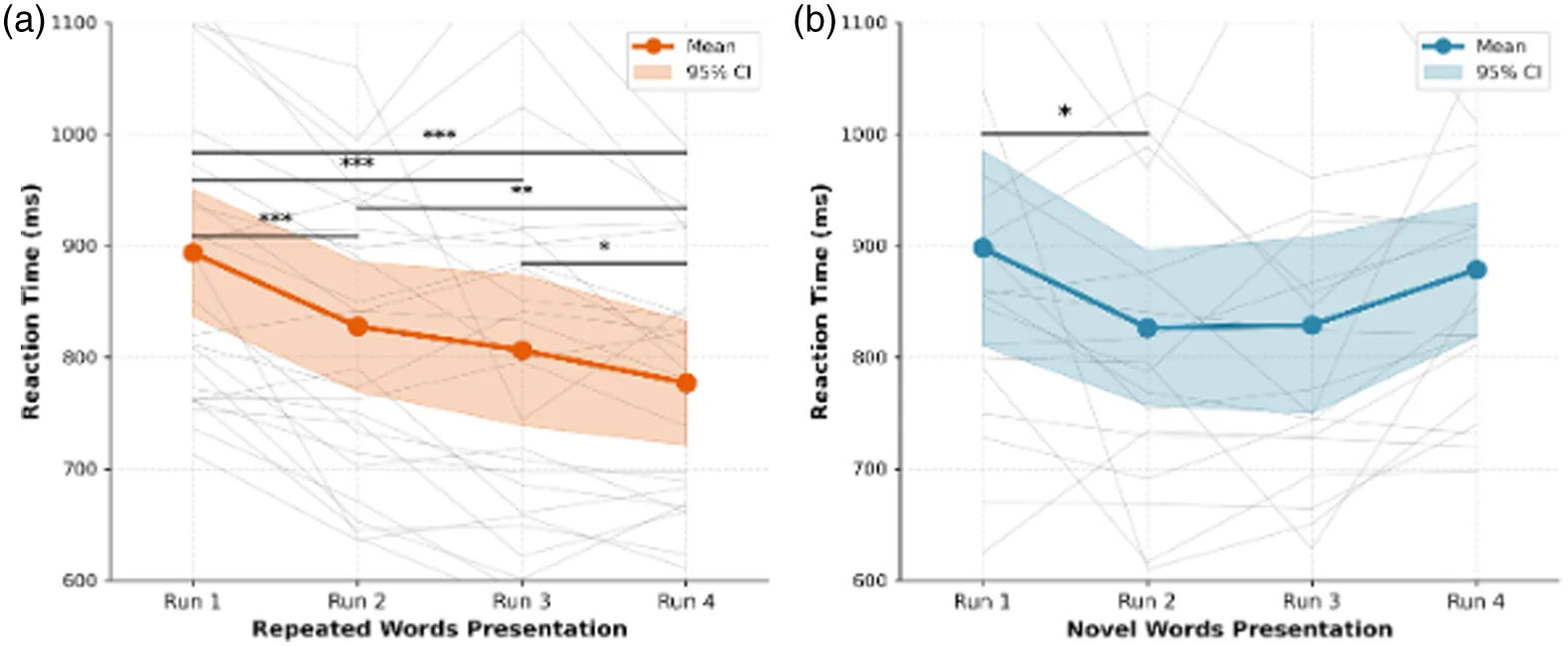
Response times (RTs) across four experimental runs for repeated and novel words. (a) Repeated words showed significant repetition priming effects, with reaction times progressively decreasing from run 1 to run 4 (run 1 versus run 2: p<.001; run 1 versus run 3: p<.001; run 1 versus run 4: p<.001). (b) Novel words showed a significant decrease only between run 1 and run 2 (p<.05), with no significant differences in subsequent runs. Colored markers represent mean reaction times; shaded regions indicate 95% confidence intervals. Gray lines show individual participant trajectories. *p<.05, ***p<.001.

### 3.2 fNIRS Unimodal Results

The GLM analysis, corrected for multiple comparisons using the Benjamini–Hochberg^61^ false-discovery rate (p-FDR<.05), identified channels with significant repetition-related differences in HbDiff. Repeated words in run 1 elicited significantly greater hemodynamic response amplitudes than in run 4 in channels positioned over the left IFG and left STG, including channel 54 (t=2.09, p-FDR=.042, d=0.40), channel 33 (t=2.23, p-FDR=.037, d=0.43), and channel 34 [t=2.89, p-FDR=.030, d=0.56; Fig. 5(a)]. By contrast, repeated words in run 4 produced significantly greater activation in the right supramarginal gyrus (SMG), with a repetition-related increase observed in channel 76 [t=−2.12, p-FDR=.043, d=0.41; Fig. 5(a)]. No additional channels survived FDR correction. Figure 5(b)–5(g) illustrate the trajectory of hemodynamic responses across runs for these significant regions. In the left IFG [Fig. 5(b)], repeated words showed a progressive decline in HbDiff beta values from run 1 (M=0.42, SD=0.18) to run 4 (M=0.15, SD=0.15), reflecting repetition reduction (p<.001). Novel words in the same region [Fig. 5(c)] exhibited no significant change across runs (p>.1). A similar pattern was observed in the left STG: repeated words [Fig. 5(d)] demonstrated significant repetition reduction from run 1 (M=0.48, SD=0.20) to run 4 (M=0.22, SD=0.18) (p<.001), whereas novel words [Fig. 5(e)] showed no significant modulation (p>.1). By contrast, repeated words in the right SMG [Fig. 5(f)] showed a significant increase in HbDiff beta values from run 1 (M=0.22, SD=0.15) to run 4 (M=0.37, SD=0.15), reflecting repetition enhancement (p<.001), whereas novel words in this region [Fig. 5(g)] showed no significant modulation across runs (p>.1).

**Fig. 5:**
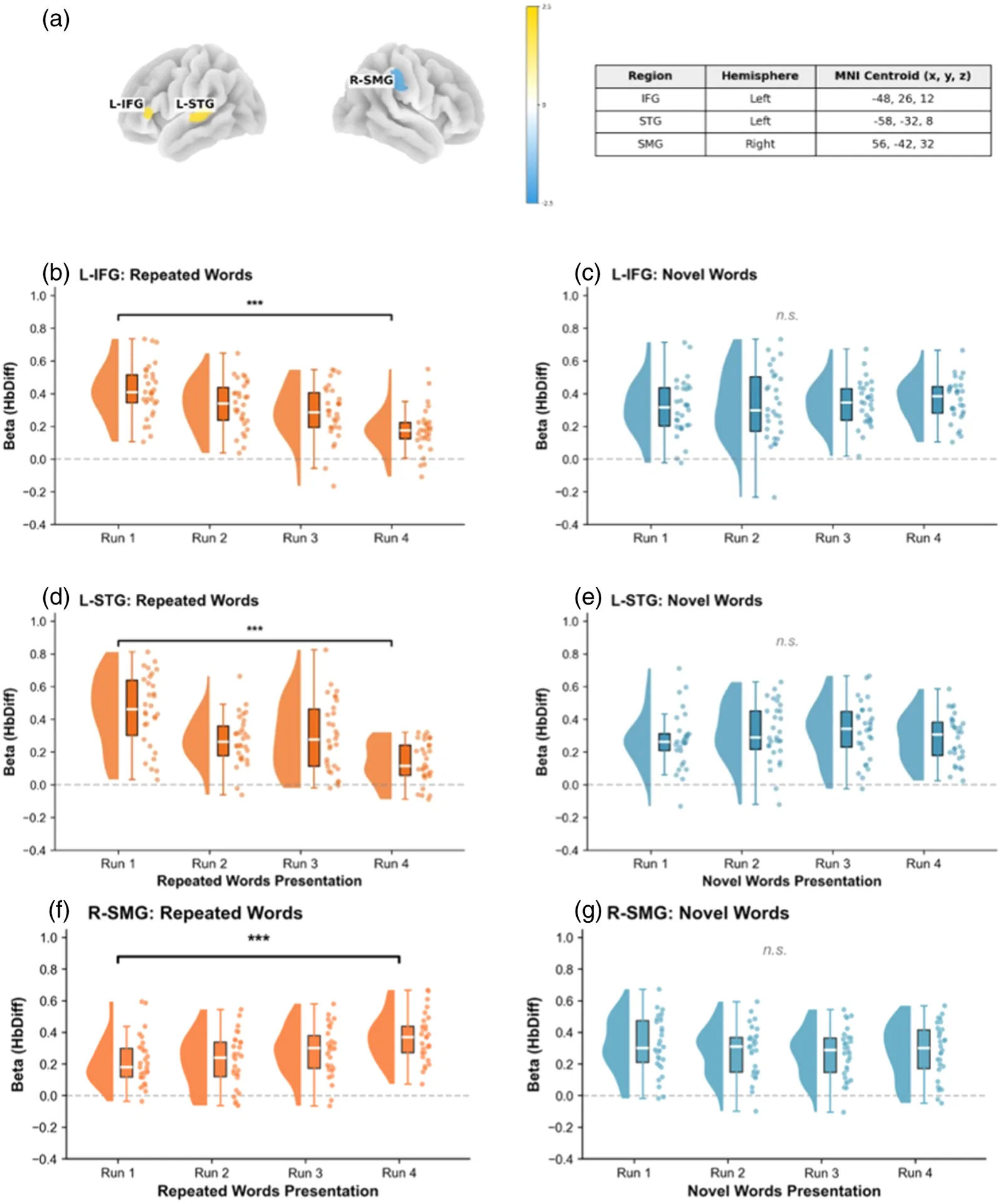
(a) Brain regions showing significant activation differences between run 1 and run 4 for repeated words. The table summarizes the MNI coordinates of each significant cluster. Yellow clusters indicate greater activation in run 1, and blue cluster indicates greater activation in run 4. Statistical maps thresholded at p-FDR<0.05. L-IFG, left inferior frontal gyrus; L-STG, left superior temporal gyrus; R-SMG, right supramarginal gyrus. (b) Mean HbDiff β values in L-IFG across four presentations of repeated words. (c) Mean HbDiff β values in L-IFG across four presentations of novel words. (d) Mean HbDiff β values in L-STG across four presentations of repeated words. (e) Mean HbDiff β values in L-STG across four presentations of novel words. (f) Mean HbDiff β values in R-SMG across four presentations of repeated words. (g) Mean HbDiff β values in R-SMG across four presentations of novel words. Dots represent individual participants (n=30). *p<.05, ***p<.001.

### 3.3 EEG Unimodal Results

#### 3.3.1 EEG whole-scalp results

Spatio-temporal cluster-based permutation tests (10,000 permutations) comparing the first and fourth presentations of repeated words revealed one significant cluster over frontal and fronto-central electrodes (Fp1, Fp2, AF3, AF4, Fz, F4, FC1, FC2, FC6; 324 to 484 ms; p=.0005, cluster-corrected; d=1.06), with the fourth presentation eliciting more negative amplitudes than the first (run 1: M=−0.30  μV; run 4: M=−0.95  μV; Fig. 6). The effect was maximal at Fz (−1.02  μV), Fp1 (−0.99  μV), and FC2 (−0.91  μV). The bilateral frontal distribution and 300 to 500 ms latency is consistent with the FN400-related response.^12^^,^^24^ A linear trend analysis confirmed that negativity increased progressively across all four runs (Run1=−0.30, Run2=−0.85, Run3=−0.93, Run4=−0.95  μV; linear contrast: t=−4.38, p=.0003, d=0.96, Fig. S1 in the Supplementary Material). No adjacent-run comparisons reached significance (all ps>.10), indicating gradual accumulation. For novel words, no significant ERP differences were found (ps>.1). No significant clusters were observed in the 500 to 800 ms window (all ps>.14). Specifically, no evidence of late positive complex (LPC) or P600-like enhancement was detected in any scalp region (ps>.1).

**Fig. 6:**
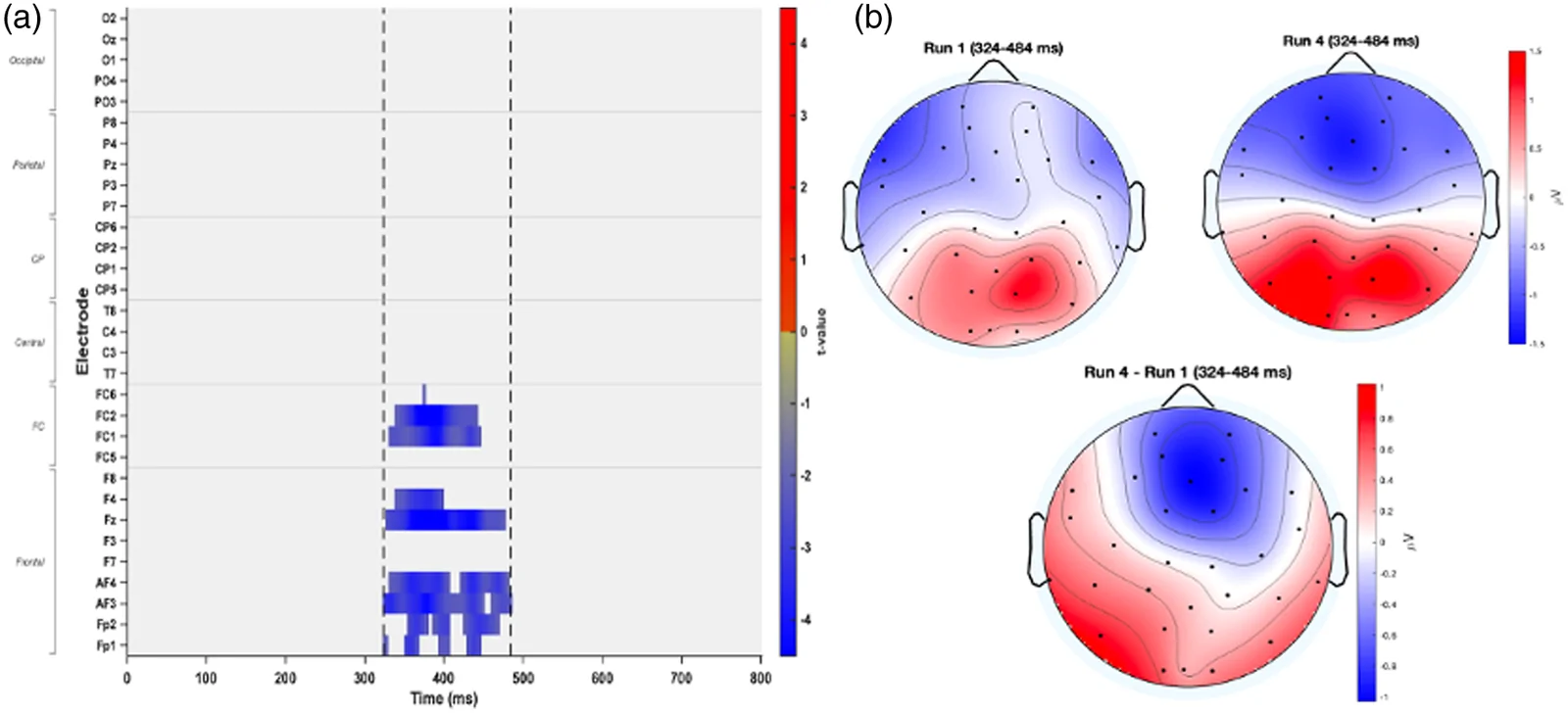
Spatio-temporal extent and scalp topography of the significant cluster-corrected ERP effect (run 4 to run 1) for repeated words. (a) Colored cells indicate electrode–time point combinations within the significant cluster identified by spatio-temporal cluster-based permutation testing (p=.0005, corrected; 10,000 permutations). Blue shading reflects t-values (negative = run 4 more negative than run 1). Dashed vertical lines mark the cluster boundaries (324 to 484 ms). The effect was concentrated over frontal and fronto-central electrodes (Fp1, Fp2, AF3, AF4, Fz, F4, FC1, FC2, FC6), consistent with the FN400-related response. Gray regions indicate nonsignificant electrode–time combinations. (b) Scalp topographies of mean amplitude averaged over the FN400 cluster window (324 to 484 ms).

#### 3.3.2 Time-frequency results

The cluster-based permutation test identified one significant cluster (p=.0014, cluster-corrected; 10,000 permutations), spanning 8 to 25 Hz (alpha and beta bands) in a late time window (520 to 800 ms; 994 time-frequency points), Fig. 7. Within this cluster, spectral power was reduced relative to baseline in run 1 (M=−1.22  dB, SD=0.76) but approached baseline levels by run 4 (M=−0.28  dB, SD=0.88), yielding a mean ERSP difference of 0.93 dB. This repetition-related power enhancement was evident in both the alpha band (8 to 13 Hz; run 1: M=−2.11  dB, SD=1.55; run 4: M=−0.97  dB, SD=1.39; t=4.51, p=.0002, d=1.04) and the beta band (13 to 25 Hz; run 1: M=−0.92  dB, SD=0.73; run 4: M=−0.04  dB, SD=0.79; t=6.16, p<.0001, d=1.41). No significant clusters were observed in the theta band (4 to 7 Hz), in any earlier time window, or for novel words (all cluster ps>.10).

**Fig. 7:**
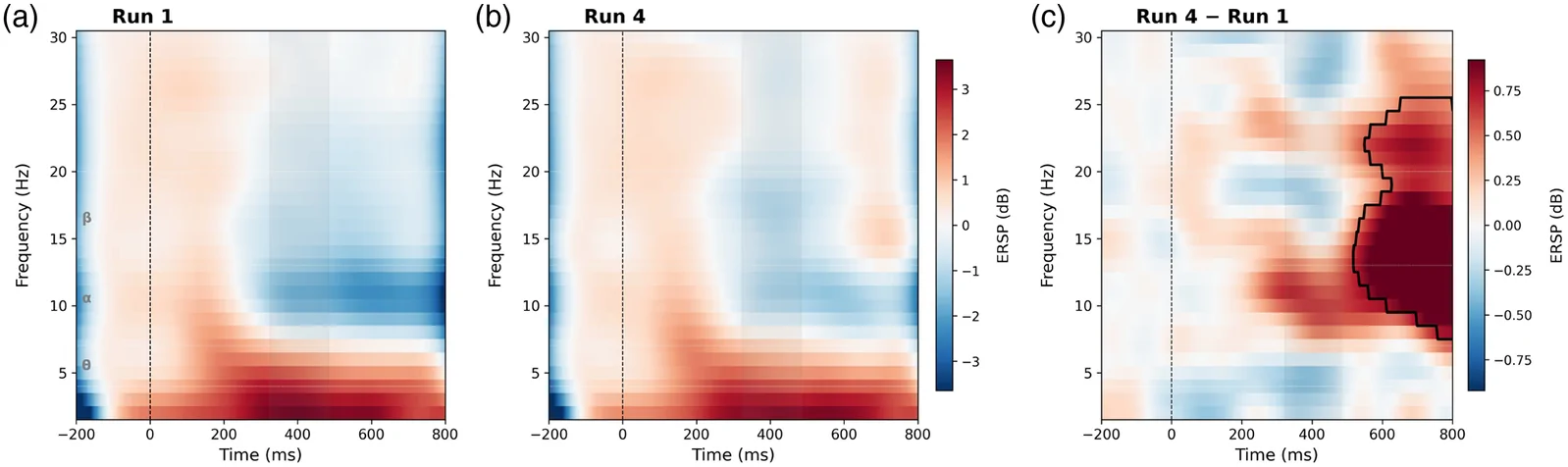
Time-frequency representations of repeated words for run 1 and run 4. (a) Event-related spectral perturbation (ERSP) for run 1, averaged across all electrodes. (b) ERSP for run 4. ERSP was computed as the decibel change relative to the pre-stimulus baseline (−200 to 0 ms) using Morlet wavelet decomposition (2 to 30 Hz, cycles=frequency/2). Panels (a) and (b) share the same color scale. Warm colors indicate power increases and cool colors indicate power decreases relative to baseline. (c) ERSP difference (run 4 to run 1). The black contour outlines the significant cluster identified by a cluster-based permutation test (10,000 permutations, p=.0014, cluster-corrected), spanning 8 to 25 Hz (alpha and beta bands) in a late time window (520 to 800 ms). Within this cluster, run 4 showed significantly greater power than run 1 (mean t=3.61), indicating repetition-related enhancement across the alpha–beta frequency range. Dashed vertical lines indicate stimulus onset (0 ms). Gray shading marks the FN400 time window (324 to 484 ms). White dotted horizontal lines indicate frequency band boundaries (theta, 4 to 7 Hz; alpha, 8 to 13 Hz; beta, 13 to 25 Hz). No significant clusters were observed for novel words.

### 3.4 Classification Results

The fNIRS-only classifier, based on regional hemodynamic features, achieved a mean accuracy of 65.42%, with individual accuracies ranging from 37.5% to 100% (4 of 30 participants achieved 100%). This performance was significantly above the *a priori* chance level of 50% according to a 1000-iteration permutation test (p=.022).

The EEG-only classifier, which incorporated ERP amplitudes (early, middle, and late time windows) and time–frequency features (theta, alpha, beta power), achieved a mean LOSO accuracy of 60.42%, with individual accuracies ranging from 37.5% to 100% (3 of 30 participants achieved 100%). This performance was significantly above chance (p=.028).

The multimodal classifier, which integrated fNIRS hemodynamic features, EEG-derived ERP and time–frequency measures, and cross-modal fNIRS–EEG correlation feature indices reduced to the top 20 principal components, achieved the highest decoding performance among the three models. The classifier achieved a mean LOSO accuracy of 72.50%, with individual accuracies ranging from 50% to 100% (6 of 30 participants achieved 100%). Performance was significantly above chance (p=.005). Paired t-tests comparing individual LOSO accuracies revealed that the multimodal classifier significantly outperformed the fNIRS-only model (t(29)=2.45, p=.021, d=0.45) and the EEG-only model (t(29)=3.12, p=.004, d=0.57).

To characterize the spatial distribution of the multimodal classifier weights, we visualized the absolute coefficients of the linear SVM for the three *a priori* regions of interest: the left inferior frontal gyrus (IFG), the left superior temporal gyrus (STG), and the left ventral occipitotemporal cortex [vOT; Fig. 8(a)]. The largest peak absolute coefficient magnitude was observed in the left IFG (2.90), followed by the left STG (2.53) and the left vOT (1.98). These values summarize the relative model-weight patterns across the feature classes included in the multimodal classifier, namely, fNIRS hemodynamic measures, EEG ERP and spectral features, and cross-modal correlation indices. At the feature-class level, fNIRS features (HbDiff mean and SD) exhibited the largest absolute coefficient magnitudes overall, followed by EEG features (ERP and spectral power), whereas cross-modal correlation features also received nonzero model weights [Fig. 8(b)]. Because predictors in multimodal EEG–fNIRS data are likely to be correlated, coefficient magnitudes may be shared or redistributed across related features. These results are therefore presented as descriptive model-weight patterns and should not be interpreted as estimates of the unique physiological importance or independent predictive contribution of individual features, feature classes, or ROIs.

**Fig. 8:**
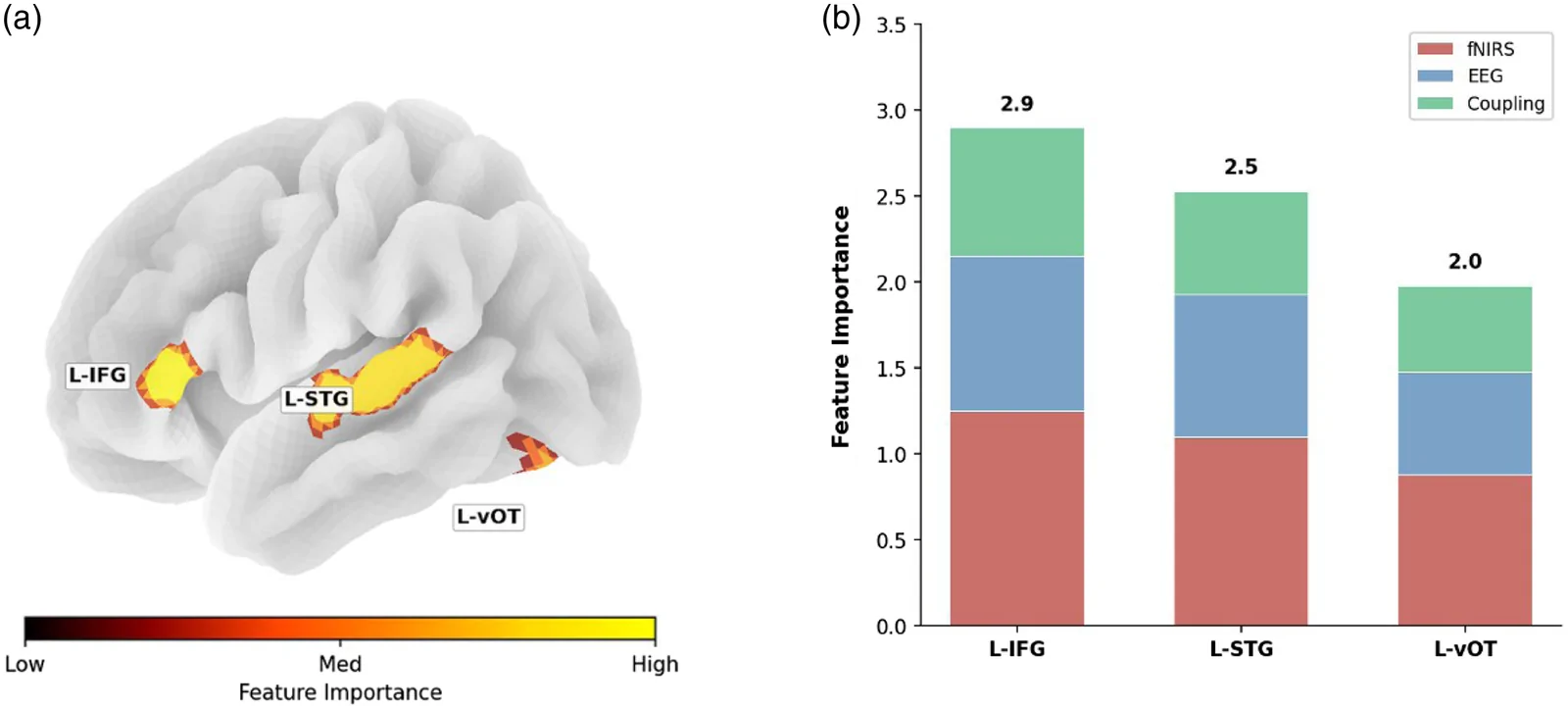
Spatial distribution and modality decomposition of classifier feature importance. (a) Warm colors denote vertices with higher classifier feature-importance values on the cortical surface. ROI labels indicate the left inferior frontal gyrus (L-IFG), left superior temporal gyrus (L-STG), and left ventral occipitotemporal cortex (L-vOT). Gyri (light gray) and sulci (dark gray) reflect FreeSurfer curvature for anatomical reference. (b) Stacked bar chart showing the decomposition of feature importance by modality for each ROI. The left IFG showed the highest total importance (2.9), followed by left STG (2.5) and left vOT (2.0). Across all ROIs, fNIRS features (HbDiff mean and SD) contributed most strongly to classification, followed by EEG features (ERP and spectral power), with cross-modal correlation features providing additional discriminative information.

The ablation analysis compared four models using the same fold-wise preprocessing, linear SVM, and LOSO procedure: fNIRS only, EEG only, direct integration of fNIRS and EEG features without the cross-modal correlation features, and the full multimodal model. The direct-integration model achieved an accuracy of 69.17%, which was higher than the fNIRS-only model (65.42%) and the EEG-only model (60.42%). Adding the cross-modal correlation features further increased accuracy to 72.50%. Second, we conducted a six-feature-matched analysis. The fNIRS model included its six prespecified features, the EEG model included six EEG features, and the multimodal model included three fNIRS and three EEG features. Feature selection was performed exclusively within the training data of each LOSO fold. Under this matched-dimensionality comparison, the multimodal model achieved 68.75% accuracy, compared with 65.42% for fNIRS and 57.92% for EEG. Thus, the tested multimodal model remained the most accurate when feature dimensionality was matched.

To assess whether repetition-related information was detectable at earlier stages of exposure, we conducted additional decoding analyses contrasting run 1 with run 2 and with run 3. Across unimodal EEG, unimodal fNIRS, and multimodal classifiers, decoding accuracy for both contrasts did not exceed chance based on permutation testing (all ps>.05). Thus, reliable discrimination between novel and repeated items emerged only for the run 1 versus run 4 contrast, indicating that repetition-related neural signatures became decodable primarily after extended temporal separation and repeated exposure.

To examine whether behavioral learning gains were associated with multimodal classification performance, we correlated RT change percentages with LOSO classification accuracy. Across the 26 participants, RT change was significantly associated with multimodal classification accuracy (Pearson’s r=−0.52, p=.007, 95%CI[−0.76,−0.17]). The association was similar using Spearman rank correlation (ρ=−0.49, p=.011). The maximum Cook’s distance was 0.12, below the conventional 4/N threshold of 0.154, indicating that no individual observation was strongly influential. Leave-one-participant-out sensitivity analyses produced Pearson correlations ranging from −0.43 to −0.60, with the negative association remaining significant in all iterations.

## 4 Discussion

The present study provides a multilevel account of repetition-based language learning by integrating behavioral measures with simultaneous EEG and fNIRS recordings in adults, with the explicit goal of establishing a multimodal neuroimaging and machine learning framework. Repeated word exposure across extended temporal separations yielded robust behavioral facilitation alongside coordinated changes at distinct levels of neural organization. Critically, these changes did not conform to a uniform pattern of neural cost reduction as predicted by dominant accounts of repetition learning.^4^^,^^5^ Instead, they revealed a temporally structured cascade of functionally distinct adaptations, suggesting that durable word learning is a multilevel transformation rather than a unitary mechanism of neural attenuation.

### 4.1 Coordinated Multilevel Signatures of Repetition-Based Language Learning

The central finding of this study is that repetition-based word learning manifests as a coordinated pattern of neural adaptation operating across distinct levels of neural organization within brain regions canonically associated with reading and language. At the behavioral level, repeated words showed a progressive 105 ms reduction in response times across four presentations, whereas novel words showed only modest and nonprogressive change, ruling out general practice effects and confirming genuine learning from prior exposure.^18^^,^^20^ At the level of event-locked neural responses, EEG captured a progressive FN400 enhancement over frontal and fronto-central electrodes, consistent with the incremental strengthening of familiarity-based recognition. The frontal distribution of this effect is distinct from the more centro-parietal distribution of the classical N400, which has been broadly linked to lexico-semantic access,^25^^,^^69^ and is instead consistent with the FN400 component identified in recognition memory studies.^12^^,^^24^^,^^26^ This distinction suggests that what accumulates with long-lag repetition is not primarily ease of semantic access but rather the strength of a familiarity signal marking the stimulus as previously encountered, a process that may be specifically impaired in children with developmental dyslexia, who show atypical ERP responses to repeated words even when semantic access is intact.^70^ The largest amplitude change occurred between the first and second presentations with gradual asymptote thereafter, closely mirroring the behavioral RT facilitation.

At the level of oscillatory dynamics, time-frequency analysis revealed a significant cluster of attenuation of alpha–beta suppression (8 to 25 Hz, 520 to 800 ms, p=.0014, cluster-corrected), emerging after the FN400 window and suggesting a post-recognition consolidation process in which newly strengthened word representations are stabilized through coordinated oscillatory activity. Alpha-band enhancement has been linked to representational stabilization and reduced reliance on externally driven processing,^28^^,^^71^ whereas beta-band enhancement has been associated with top-down predictive processing and memory reactivation.^72^ Together with the FN400, these findings suggest a two-stage process within each repeated word: rapid familiarity-based recognition followed by oscillatory representational consolidation. Identifying whether this two-stage architecture is disrupted in children with reading difficulties is a key question motivating translation of the present protocol.

At the hemodynamic level, fNIRS revealed reduced hemodynamic responses in the left inferior frontal and superior temporal regions, core components of the reading and language network,^73^ indicating increased metabolic efficiency with word learning,^4^ alongside increased activation in the right supramarginal gyrus, suggesting continued engagement of integrative and consolidation-related processes.^8^^,^^10^ The involvement of these specific regions is noteworthy: left inferior frontal and superior temporal cortices are consistently reported as hypoactivated in developmental dyslexia during reading and phonological processing tasks,^16^ suggesting that atypical learning-related metabolic reorganization in these regions may be a sensitive marker of reading difficulty.

The temporal alignment of these effects is informative. The FN400 showed its largest amplitude change between the first and second presentations and approached asymptote by the third, closely paralleling the behavioral RT data. The alpha–beta enhancement was concentrated in a later window (520 to 800 ms), temporally following the FN400 effect and suggesting a post-recognition consolidation stage. At the hemodynamic level, the fNIRS suppression in left frontotemporal regions and enhancement in the right SMG emerged as cumulative effects across all four presentations. This pattern is consistent with a cascading architecture in which rapid familiarity signaling precedes and drives both oscillatory stabilization and metabolic reorganization, with hemodynamic signals reflecting the accumulated consequences of repeated neural computations. This cascading structure defines specific neural targets for investigation in pediatric populations.

The coexistence of suppression and enhancement across regions and modalities challenges unitary accounts of repetition learning predicting uniform decreases in neural activity^4^^,^^5^ and has important implications for how neuroimaging is used to characterize reading and language development. The dissociable pattern observed here, in which core processing regions become more efficient while other regions and frequency bands increase engagement, suggests that region-specific and multimodal analysis is necessary for a complete characterization of learning-related change. This distinction has important implications for clinical neuroimaging: studies comparing children with and without reading difficulties may misinterpret atypical enhancement as dysfunction rather than as a marker of a different stage of learning, underscoring the need for bidirectional analysis in developmental populations.

### 4.2 Nonredundancy of Electrophysiological and Hemodynamic Signatures and Implications for Phenotyping

The multimodal classification results provide the most direct evidence that repetition-based word learning engages partially independent processes at different physiological levels and offer the strongest argument for a multimodal approach to phenotyping language learners. The multimodal classifier (72.50% accuracy) significantly outperformed both the fNIRS-only (65.42%) and EEG-only (60.42%) classifiers, and this improvement cannot be attributed to simply having more features, as the multimodal model was trained with PCA-reduced feature sets under the same leave-one-subject-out cross-validation framework. This nonredundancy indicates that repetition learning produces partially independent adaptations at different levels of neural organization, each capturing a different facet of the representational transformation, and therefore, no single modality can fully characterize the learning process or the individual differences within it.

The feature-importance analysis illuminated the basis of this complementarity. Across all three regions of interest, fNIRS features contributed most strongly to classification, consistent with the notion that hemodynamic signals integrate across rapid electrophysiological events to provide a cumulative index of regional metabolic adaptation. EEG features captured the temporal dynamics of predictive updating and familiarity signaling that unfold within hundreds of milliseconds. The additional contribution of cross-modal fNIRS–EEG correlation features is particularly noteworthy as it indicates that learning modifies not only the amplitude of neural responses at each level but also the coupling between electrical activity and its metabolic consequences,^74^^,^^75^ providing information not fully captured by either signal alone,^76^^,^^77^ a dimension of neural organization that may be specifically disrupted in neurodevelopmental reading and language disorders.

Critically, individual differences in multimodal classification accuracy correlated with behavioral learning magnitude, demonstrating that the neural signatures captured by this framework track meaningful variation in language learning capacity across individuals. This finding is foundational for the translational goal of the present work: if multimodal decoding accuracy indexes individual learning capacity in adults, it may serve as a sensitive marker for identifying children at risk for reading and language difficulties, or for tracking the neural effects of language interventions over time.

### 4.3 Theoretical Implications

The present findings have implications for how repetition-based language learning is conceptualized within existing theoretical frameworks. Predictive coding accounts propose that repetition reduces prediction error, leading to attenuated neural responses,^6^^,^^78^ and the FN400 and frontotemporal hemodynamic suppression observed here are broadly compatible with this account. However, predictive coding alone does not fully account for the concurrent enhancements observed in other signals and regions nor the temporal dissociation between ERP and oscillatory effects, suggesting that repetition-based language learning engages additional processes beyond prediction error reduction. The two-stage cascade proposed above, rapid familiarity recognition followed by oscillatory consolidation and metabolic reorganization, may help reconcile the broader literature on repetition suppression and enhancement^10^^,^^11^ and provides a more tractable neural model for understanding where and how language learning breaks down in developmental disorders.

### 4.4 Toward a Multimodal Framework for Phenotyping Reading and Language Development

The present findings motivate several directions for translating the multimodal fNIRS-EEG framework to the study of reading and language difficulties in children. First, disruptions at different stages of the learning cascade, rapid familiarity signaling versus post-recognition consolidation, may map onto different profiles of reading and language difficulty, with implications for targeted intervention. Second, individual differences in fNIRS-EEG coupling strength may predict longer-term retention or consolidation efficiency,^79^ providing a candidate neural marker for children who fail to consolidate word-form representations despite repeated exposure, a hallmark of developmental dyslexia. Third, extending the present approach to tasks explicitly engaging phonological processing and orthographic learning would more directly probe the mechanisms underlying reading acquisition and its disorders. Methodologically, the present study establishes concurrent fNIRS-EEG as a particularly well-suited platform for pediatric research. Its tolerance of movement, minimal electromagnetic interference, participant comfort, and compatibility with extended task durations address the practical constraints of neuroimaging with children, especially those with attention and language difficulties for whom fMRI is often impractical. The machine learning decoding framework developed here provides a principled approach to extracting individual-level markers of language learning capacity from multimodal data and is directly adaptable to clinical and educational research settings.

### 4.5 Limitations and Future Directions

First, the cross-modal correlation features quantify feature-level statistical correspondence between fNIRS and EEG measures rather than temporally modeled physiological neurovascular coupling. Because each correlation was estimated from only six trials and did not account for the delayed hemodynamic response, the estimates may be sensitive to sampling variability and should be interpreted as exploratory. Second, in the current design, repeated and novel conditions differed in prevalence and predictability; some condition-related effects may therefore reflect expectancy or task-context differences in addition to repetition history. Third, because short-separation channels were not available, superficial scalp blood flow could not be directly measured or regressed out. Although PCA-based spatial filtering attenuated spatially widespread physiological fluctuations, it cannot fully separate cortical activity from extracerebral signals, particularly in frontal regions. Accordingly, the left frontal fNIRS effects should be interpreted cautiously as they may retain superficial or systemic physiological contributions. Future studies should incorporate short-separation channels and concurrent systemic physiological measurements. Finally, the primary use of HbDiff limits the physiological specificity of the present findings because potentially informative differences in the direction and relationship of HbO and HbR were not examined separately. Future studies should analyze both chromophores independently to provide a more complete characterization of hemodynamic responses and neurovascular coupling.

## 5 Conclusion

The present findings provide a multilevel account of repetition-based language learning in adults, demonstrating that behavioral facilitation is accompanied by coordinated yet nonredundant adaptations across electrophysiological and hemodynamic levels of the reading and language network. By combining simultaneous fNIRS-EEG with multimodal machine learning decoding, this study captures rapid familiarity-based recognition, post-recognition representational stabilization, and slower metabolic reorganization, each indexing a distinct and nonredundant facet of how durable word representations are built. Critically, individual differences in multimodal classification accuracy track behavioral learning gains, establishing a foundation for using this framework to phenotype individual differences in language learning capacity. These findings position simultaneous fNIRS-EEG combined with multimodal machine learning as a powerful and practically translatable platform for characterizing the neural basis of reading and language development and for identifying markers of reading and language difficulties in pediatric populations.

## Supplementary Material

10.1117/1.NPh.13.4.045001.s01

## Data Availability

All data (raw and processed EEG, fNIRS, behavioral) and analysis code are publicly available on the Open Science Framework at https://osf.io/gkr7h/overview?view_only=fc8146cf98374343929ddaef9e5f703f.
