# Supplementary material for: Multiscale neural signatures of long-lag repetition learning: a simultaneous fNIRS–EEG study
Source: Neurophotonics. 2026 Oct 16;13(4):045001. doi: 10.1117/1.NPh.13.4.045001 (PMC13673251; doi:10.1117/1.NPh.13.4.045001)
Supplement: Supplementary file 1 [file NPh_013_045001_SD001.pdf]

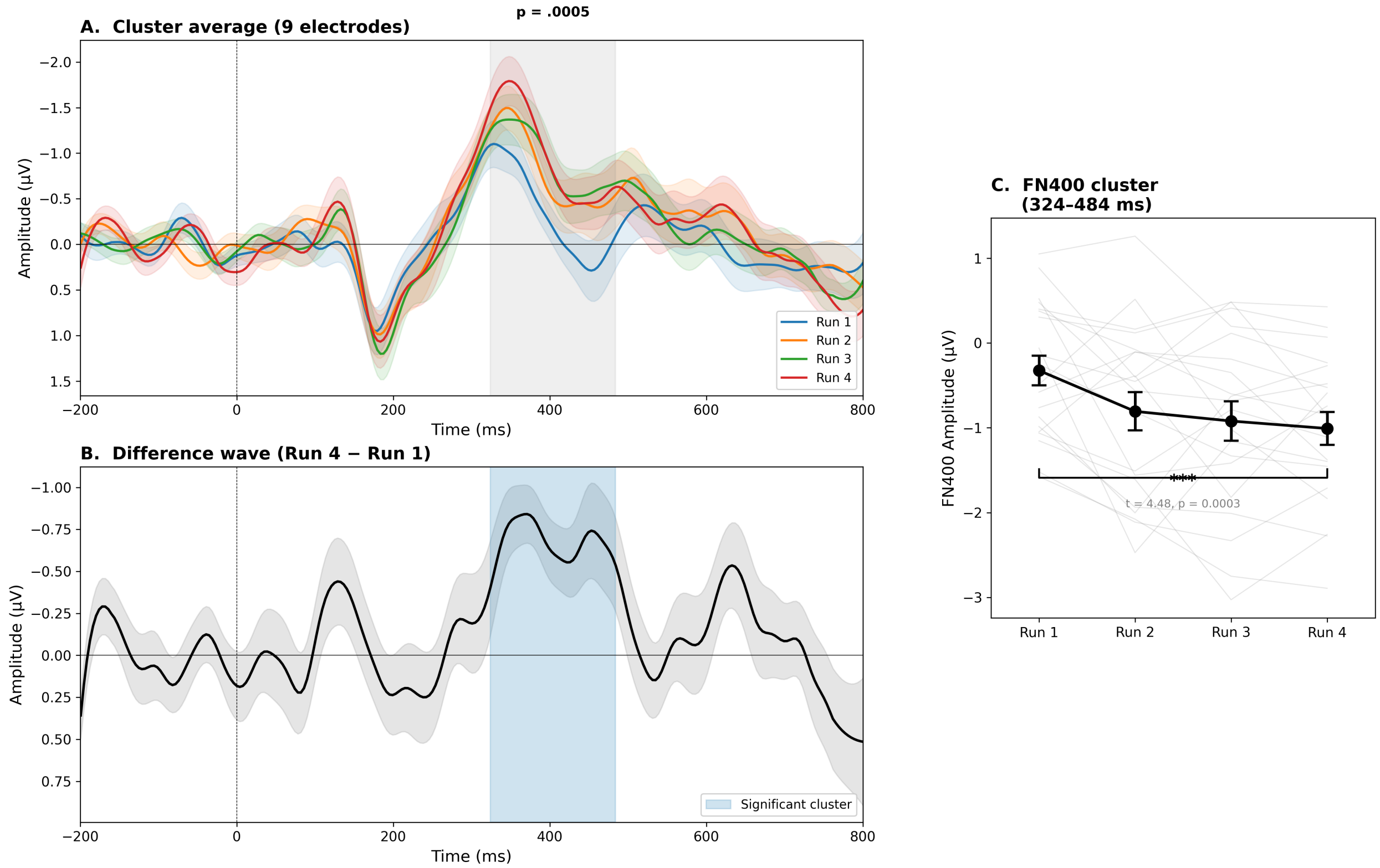


**Figure S1.** FN400 repetition effect across four presentations of repeated words. (A) Grand-averaged ERP waveforms averaged across the nine electrodes within the significant cluster (Fp1, Fp2, AF3, AF4, Fz, F4, FC1, FC2, FC6) for Runs 1–4. Shaded areas represent ± 1 SEM. Gray shading indicates the significant cluster interval (324–484 ms). (B) Difference waveform (Run 4 − Run 1) averaged across the same nine cluster electrodes. Blue shading marks the significant cluster window. The negative deflection within this window reflects the progressive FN400 enhancement with repetition. (C) Mean amplitude within the FN400 cluster (324–484 ms; 9 electrodes) across four runs. Gray lines represent individual participants; black circles represent the group mean ± SEM. The bracket indicates the paired comparison between Run 1 and Run 4 (***p < .001). Negative is plotted upward in panels A and B.

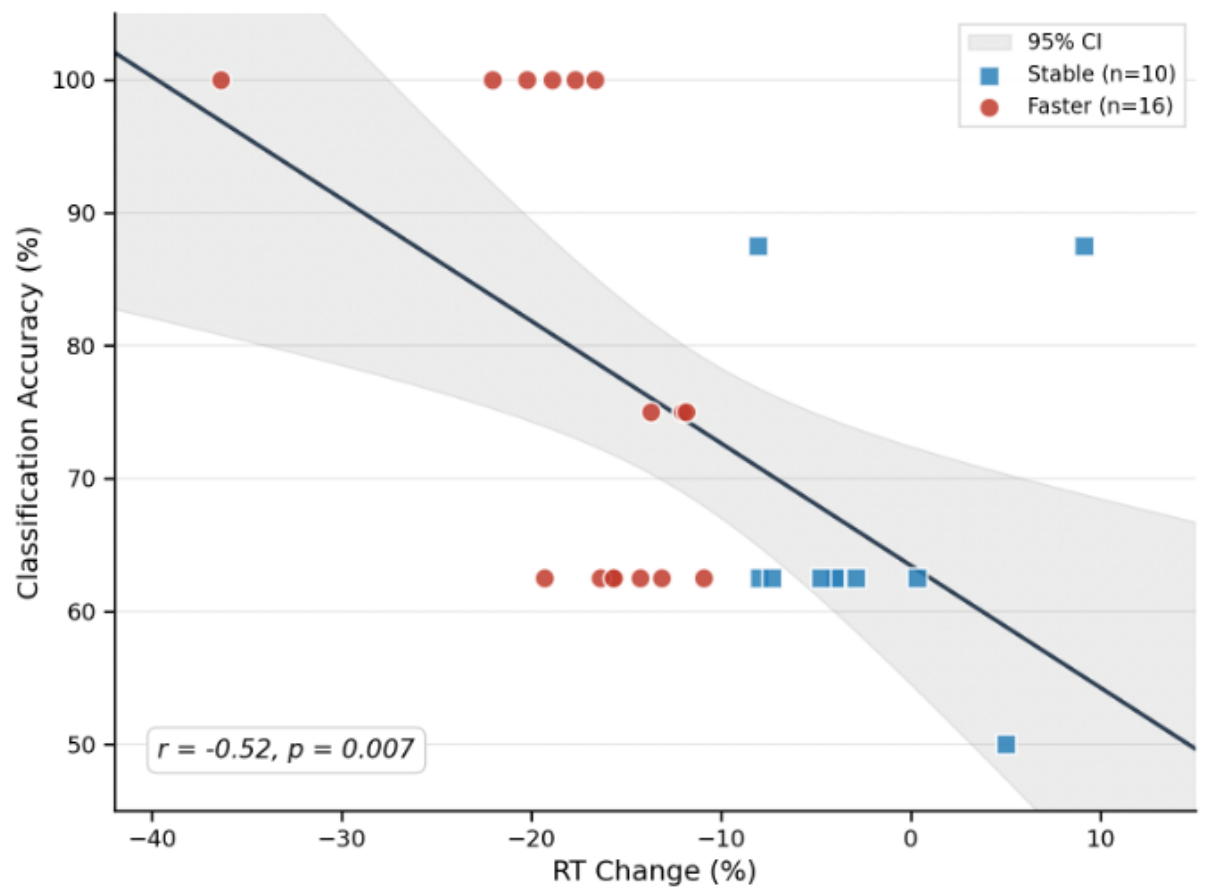


**Fig. S2.** Scatter plot showing the relationship between behavioral reaction time (RT) change and multimodal classification accuracy. RT change was calculated as (Run 4 − Run 1) / Run 1 × 100%, with more negative values indicating greater RT reduction and stronger behavioral learning. Each point represents an individual participant. Participants were categorized post hoc as Faster learners (>10% RT reduction; red circles) or Stable learners (<10% RT change; blue squares). The solid line represents the linear regression fit, and the shaded region indicates the 95% confidence interval.
